# Integrated Guidance and Control Using Model Predictive Control with Flight Path Angle Prediction against Pull-Up Maneuvering Target

**DOI:** 10.3390/s20113143

**Published:** 2020-06-02

**Authors:** Jongho Park, Youngil Kim, Jong-Han Kim

**Affiliations:** 1 Department of Military Digital Convergence, Ajou University, Suwon 16499, Korea; parkjo05@ajou.ac.kr; 2 Department of Aerospace Engineering, Seoul National University, Seoul 08826, Korea; calav3@snu.ac.kr; 3 Department of Electronic Engineering, Kyung Hee University, Yongin 17104, Korea

**Keywords:** integrated guidance and control, terminal homing, model predictive control, convex optimization

## Abstract

Integrated guidance and control using model predictive control against a maneuvering target is proposed. Equations of motion for terminal homing are developed with the consideration of short-period dynamics as well as actuator dynamics of a missile. The convex optimization problem is solved considering inequality constraints that consist of acceleration and look angle limits. A discrete-time extended Kalman filter is used to estimate the position of the target with a look angle as a measurement. This is utilized to form a flight-path angle of the target, and polynomial fitting is applied for prediction. Numerical simulation including a Monte Carlo simulation is performed to verify the performance of the proposed algorithm.

## 1. Introduction

Research on missile guidance and control fields is being actively conducted nowadays, as it has always been [1,2,3,4,5,6,7,8,9,10]. Traditionally, the guidance system and control system of an interceptor missile are separately designed. In general, the control system is placed inside the guidance system as an inner-loop that follows the acceleration command generated by the guidance system. Although traditional proportional navigation is effective and simple for practical implementation, its transient performance may be unsatisfactory in terminal homing due to various reasons. For example, the classical guidance and control approach of the missile shows the cascaded structure in which the outer loop and the inner loop consist of guidance system and autopilot, respectively. In terminal homing, the guidance loop bandwidth increases as the missile approaches close to the target, whereas the autopilot loop bandwidth remains the same. This may result in a poor autopilot response and even lead to instability of the missile at the end.

All of the aforementioned guidance studies [1,2,3,4,5,6,7,8,9,10], as well as advanced guidance research such as Impact Angle Control Guidance (IACG) [11,12,13], Impact Time and Angle Control Guidance (ITACG) [14], or circular navigation guidance [15] only consider some form of guidance problems. As they are combined with the control loops, most of them suffer from terminal instability issues in the vicinity of the impact. Many of these guidance laws assume simple (first-order or second-order) autopilot models in the performance analysis. However, the discrepancy from the assumed model usually results in unexpected or even divergent responses.

As a consequence, integrated guidance and control design has been developed to deal with this problem. The integration mainly results in reducing two cascaded loops into a single one and removing a lag between the commanded and achieved accelerations from guidance and autopilot, respectively. Menon and Ohlmeyer [16] applied the feedback linearization method with the help of Brunovsky’s canonical form. Then, the infinite-time horizon Linear Quadratic Regulator (LQR) was employed. Shima et al. [17] proposed a Sliding-Mode Control (SMC) approach for the derivation of integration where zero-effort miss was used to define the sliding surface. Small miss distances were achieved in stringent scenarios through simulation. Idan et al. [18] also used SMC for a missile controlled by two aerodynamic surfaces. An additional degree of freedom was used to improve the interceptor’s dynamic response. Xin et al. [19] used the θ-*D* technique to solve the nonlinear infinite-horizon integrated guidance and control problem. The method gave an approximate closed-form suboptimal feedback control with no iterative solutions. On the other hand, Vaddi et al. [20] developed a numerical approach based on the state-dependent Riccati equation solution. State-dependent system matrices were obtained by a constrained least-squares optimization problem. Kim et al. [21] introduced an explicit solution of time-varying state feedback form, which could be easily implemented onboard. Various terminal and interim constraints could be handled with the proposed approach. He et al. [22] dealt with the problem of impact angle constraint and integrated guidance and control law design for maneuvering target interception. The systematic backstepping technique was adopted where the convergence of line-of-sight rate and impact angle error were regulated by two independent virtual control laws. Wang et al. [23] considered control saturation and multiple disturbances both in the guidance loop and the control loop. The system was stabilized through the classical dynamic surface control method, and a linear matrix inequality approach was utilized for the analysis of the quadratic stability of the integrated system. Liu et al. [24] combined dynamic surface control with the Barrier Lyapunov Function (BLF) for skid-to-turn missiles. Input saturation and constraints of attack angle, sideslip angle, and velocity deflection angle were taken into consideration. Chai et al. [25] constructed a nonlinear Receding Horizon Pseudospectral Control (RHPC) scheme to generate the optimal control command. The problem of state estimation was solved by implementing a Moving Horizon Estimation (MHE) algorithm.

Among various strategies, Model Predictive Control (MPC) is adopted by several researchers. MPC is well known for its ability to handle constraints. Thus, the technique is suited for the terminal homing situation with missile constraints. Mehra et al. [26] used nonlinear MPC to handle hard constraints on the position and rates of the control surfaces. The method provided good performance for different types of coupled maneuvers. Kang et al. [27] extended nonlinear model predictive tracking control for a bank-to-turn missile. The explicit model was employed to predict future output behavior with the help of a Kalman filter. Bachtiar et al. [28] presented an MPC scheme with a nonlinear prediction model and an ellipsoidal terminal constraint. The proposed method showed a superior tracking performance compared to a linear prediction model. Li et al. [29] formulated a quadratic programming problem using a neurodynamic optimization approach. MPC was employed with linear variable inequality based a primal–dual neural network based on tracking kinematics. Bachtiar et al. [30] demonstrated a model-predictive integrated missile control design to improve control performance by commanding optimal acceleration. The control pushed the missile to be more responsive than a conventional separated guidance and autopilot system.

Pull-up is a popular evasive maneuver for surface-to-surface missiles against surface-to-air missiles. During the pull-up maneuver, the target acceleration changes in a linear fashion or stays at a nonzero constant [31]. Note that the weaving maneuver, which is another popular evasive maneuver, is not considered in this study. As the acceleration of the target is unknown to the interceptor, a flight-path angle of the target is also unknown.

In this study, integrated guidance and control using MPC with flight-path angle prediction is proposed. A short-period dynamics and guidance kinematics between a missile and a target are considered [17,21,32,33]. The optimization problem for MPC is formulated using equality and inequality constraints [34]. An acceleration constraint is considered because of the finite maneuver capability of the missile, and a look angle constraint is considered due to the limitation of a strapdown seeker’s image plane [35]. The formulated problem is solved by the primal–dual interior-point method [36,37]. With a discrete-time Extended Kalman Filter (EKF), a position of the target is estimated using a look angle from the strapdown seeker as a measurement [38,39]. The look angle is considered with a quantization effect [40]. A flight-path angle of the target is constructed and stored for polynomial curve fitting, and the obtained polynomial coefficients are utilized in MPC. Numerical simulation is performed to demonstrate the performance of the proposed algorithm. Specifically, the robustness of the proposed algorithm is verified using a Monte Carlo simulation approach.

The contributions of this study are summarized as follows. First, the proposed integrated guidance and control algorithm can handle the target missile with a pull-up maneuver, whereas the previous work [21] only deals with the stationary target. Second, from a practical standpoint, physical constraints of acceleration and look angle are considered in the problem formulation. Third, flight-path angle prediction is conducted with the help of look angle measurement from a strapdown seeker.

The paper is organized as follows. Section 2 formulates the MPC problem considered in this study. And, Section 3 shows how the predicted flight-path angle of the target is incorporated in MPC. In Section 4, numerical simulation results are presented. Finally, the conclusion is given in Section 5.

## 2. Problem Formulation

**Figure 1:**
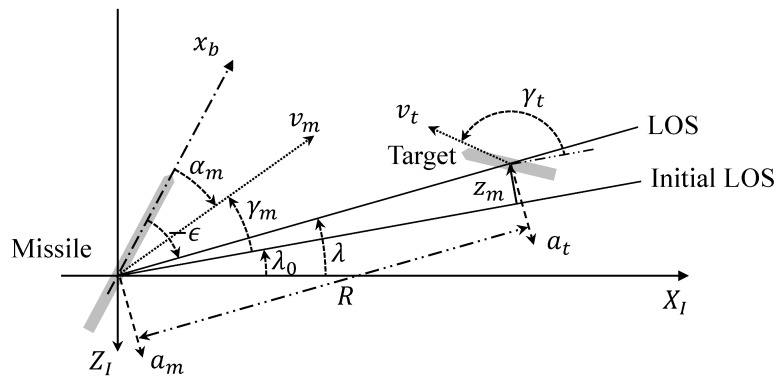
Two-dimensional engagement geometry.

A two-dimensional terminal homing problem is considered, as shown in Figure 1. In Figure 1, $X_{I}-Z_{I}$ is a reference coordinate system whose origin is located at the missile’s initial center of gravity. It is assumed that the missile is close to a collision triangle at the beginning of the terminal homing, and the missile and target deviations during the terminal homing are sufficiently small. Therefore, linearization can be performed around the initial Line-Of-Sight (LOS), $\lambda_{O}$, in Figure 1. Also, constant known speed is assumed for both the missile and the target. $x_{b}$ is a body-fixed coordinate system, and $z_{m}$ is a relative displacement between the missile and the target, normal to the initial LOS. *R* and λ are the range-to-go and LOS of the missile and the target, respectively. Furthermore, $v_{m}$, $\alpha_{m}$, and $\gamma_{m}$ are speed, angle of attack, and flight-path angle of the missile, respectively. $a_{m}$ and $a_{t}$ are accelerations of the missile and the target, respectively, normal to the LOS as shown in Figure 1. Note that gravitational force is neglected for simplicity in the engagement kinematics; however, it should be noted that the computational framework we use in this paper easily extends to the cases with gravitational force [21]. Also note that it is common that the conventional guidance solutions are first obtained without considering the gravity, and the gravity is compensated for the implementation [32].

The short-period dynamics of the missile is governed by the following equation:

(1) $$\left[\begin{array}{c} {\dot{\alpha }}_{m}\\ {\dot{q}}_{m} \end{array}\right]=\left[\begin{array}{cc} Z_{\alpha } & 1\\ M_{\alpha } & M_{q} \end{array}\right]\left[\begin{array}{c} \alpha_{m}\\ q_{m} \end{array}\right]+\left[\begin{array}{c} Z_{\delta }\\ M_{\delta } \end{array}\right]\delta_{m},$$

where $q_{m}$ is a pitch rate, $\delta_{m}$ is a control fin deflection angle, and $Z_{\alpha }$, $Z_{\delta }$, $M_{\alpha }$, $M_{q}$, and $M_{\delta }$ are corresponding dimensional derivatives of the missile. Now, $a_{m}$ can be expressed as follows:

(2) $$a_{m}=v_{m}\left({\dot{\alpha }}_{m}-q_{m}\right)=v_{m}Z_{\alpha }\alpha_{m}+v_{m}Z_{\delta }\delta_{m}.$$

The control fin actuator dynamics is modeled as a single lag system as follows:

(3) $${\dot{\delta }}_{m}=-\omega_{a}\delta_{m}+\omega_{a}\delta_{c},$$

where $\delta_{c}$ is a control command and $\omega_{a}$ is an inverse of the actuator time constant.

On the other hand, the terminal homing kinematics can be described as follows:

(4) $$\left[\begin{array}{c} {\dot{\gamma }}_{m}\\ {\dot{z}}_{m} \end{array}\right]=\left[\begin{array}{cc} 0 & 0\\ -v_{m} & 0 \end{array}\right]\left[\begin{array}{c} \gamma_{m}\\ z_{m} \end{array}\right]+\left[\begin{array}{c} -1/v_{m}\\ 0 \end{array}\right]a_{m}+\left[\begin{array}{c} 0\\ v_{t}\sin\gamma_{t} \end{array}\right],$$

where $a_{m}$ can be replaced by Equation (2). $v_{t}$ and $\gamma_{t}$ are the speed and flight-path angle of the target, respectively. Using Equation (1), Equations (3) and (4), the final equations of motion for terminal homing can be obtained as follows:

(5) $$\begin{array}{ccc} \underset{\equiv \dot{\bar{x}}}{\underset{︸}{\left[\begin{array}{c} {\dot{\delta }}_{m}\\ {\dot{\alpha }}_{m}\\ {\dot{q}}_{m}\\ {\dot{\gamma }}_{m}\\ {\dot{z}}_{m} \end{array}\right]}} & = & \underset{\equiv \bar{A}}{\underset{︸}{\left[\begin{array}{ccccc} -\omega_{a} & 0 & 0 & 0 & 0\\ Z_{\delta } & Z_{\alpha } & 1 & 0 & 0\\ M_{\delta } & M_{\alpha } & M_{q} & 0 & 0\\ -Z_{\delta } & -Z_{\alpha } & 0 & 0 & 0\\ 0 & 0 & 0 & -v_{m} & 0 \end{array}\right]}}\underset{\equiv \bar{x}}{\underset{︸}{\left[\begin{array}{c} \delta_{m}\\ \alpha_{m}\\ q_{m}\\ \gamma_{m}\\ z_{m} \end{array}\right]}}+\underset{\equiv \bar{B}}{\underset{︸}{\left[\begin{array}{c} \omega_{a}\\ 0\\ 0\\ 0\\ 0 \end{array}\right]}}\underset{\equiv \bar{u}}{\underset{︸}{\delta_{c}}}+\underset{\equiv \bar{b}}{\underset{︸}{\left[\begin{array}{c} 0\\ 0\\ 0\\ 0\\ v_{t}\sin\gamma_{t} \end{array}\right]}}. \end{array}$$

Equation (5) can be discretized with the sampling interval of $\Delta t_{c}$ as follows:

(6) $$x_{k+1}=Ax_{k}+Bu_{k}+b,$$

where $A=e^{\Delta t_{c}\bar{A}}$, $B=\left(\int_{0}^{\Delta t_{c}}e^{\tau \bar{A}}\right)\bar{B}$, and $b=\left(\int_{0}^{\Delta t_{c}}e^{\tau \bar{A}}\right)\bar{b}$.

In addition, the look angle can be defined and approximated with the small angle approximation as follows:

(7) $$\epsilon =\left(\lambda -\lambda_{0}\right)-\alpha_{m}-\gamma_{m}≃z_{m}/R-\alpha_{m}-\gamma_{m}.$$

Now, the acceleration and look angle constraints can be expressed in the matrix form.

(8) $$\underset{\equiv C_{k}}{\underset{︸}{\left[\begin{array}{ccccc} v_{m}Z_{\delta } & v_{m}Z_{\alpha } & 0 & 0 & 0\\ -v_{m}Z_{\delta } & -v_{m}Z_{\alpha } & 0 & 0 & 0\\ 0 & -1 & 0 & -1 & 1/R_{k}\\ 0 & 1 & 0 & 1 & -1/R_{k} \end{array}\right]}}\left[\begin{array}{c} \delta_{m}\\ \alpha_{m}\\ q_{m}\\ \gamma_{m}\\ z_{m} \end{array}\right]\leq \underset{\equiv D}{\underset{︸}{\left[\begin{array}{c} a_{max}\\ a_{max}\\ \epsilon_{max}\\ \epsilon_{max} \end{array}\right]}},$$

where $a_{max}$ and $\epsilon_{max}$ are the missile acceleration and look angle limits, respectively, and $R_{k}$ is the range-to-go after *k* samples, which can be calculated as follows:

(9) $$R_{k}=R_{0}-k\left(v_{m}+v_{t}\right)\Delta t_{c},$$

where $R_{0}$ is the current range-to-go.

Finally, the MPC problem is formulated as follows:

(10) $$\begin{array}{cccc} \; & \underset{u_{0},\cdots ,u_{N-1}}{\mathrm{minimize}} & \; & \sum_{k=0}^{N-1}\left(x_{k}^{T}Q_{k}x_{k}+u_{k}^{T}R_{k}u_{k}\right)+x_{N}^{T}Q_{N}x_{N}\\ \; & \mathrm{subject}\;\mathrm{to} & \; & x_{k+1}=Ax_{k}+Bu_{k}+b\\ \; & \; & \; & C_{k}x_{k}\leq D, \end{array}$$

for all k∈{0,⋯,N−1}, where $Q_{k}$ and $R_{k}$ are weighting matrices of $x_{k}$ and $u_{k}$, respectively. In Equation (10), subscript 0 indicates the current time and *N* is the number of samples. Therefore, the length of the time horizon in MPC, $t_{c}$, can be computed as follows:

(11) $$t_{c}=N\Delta t_{c}.$$

Note that *b* is an unknown matrix due to $\gamma_{t}$.

## 3. Model Predictive Control with Flight-Path Angle Prediction

### 3.1. MPC Procedure

**Figure 2:**
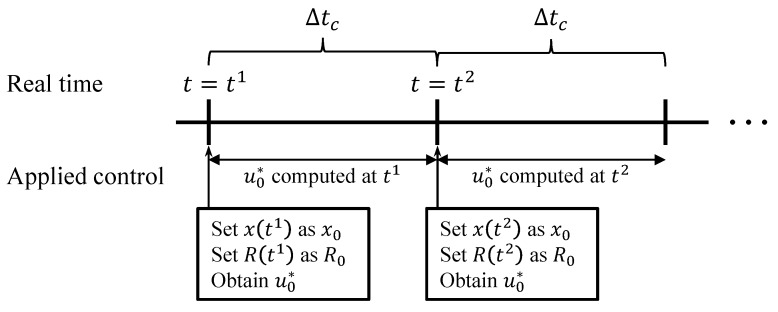
Model Predictive Control (MPC) scheme.

The MPC process for terminal homing is shown in Figure 2. At time $t^{1}$, the current state is sampled and the optimization problem in Equation (10) is computed via a numerical optimization algorithm. Among the obtained optimal control states, $u_{0}^{*},\cdots ,u_{N-1}^{*}$, only the first step, $u_{0}^{*}$, is implemented to the control fin actuator. At time $t^{2}$, the state is sampled again and the calculation is repeated starting from the new current state. Then, a new $u_{0}^{*}$ is obtained and implemented. This iterative procedure continues until the missile intercepts the target.

### 3.2. Look Angle Property: Quantization Effect

**Figure 3:**
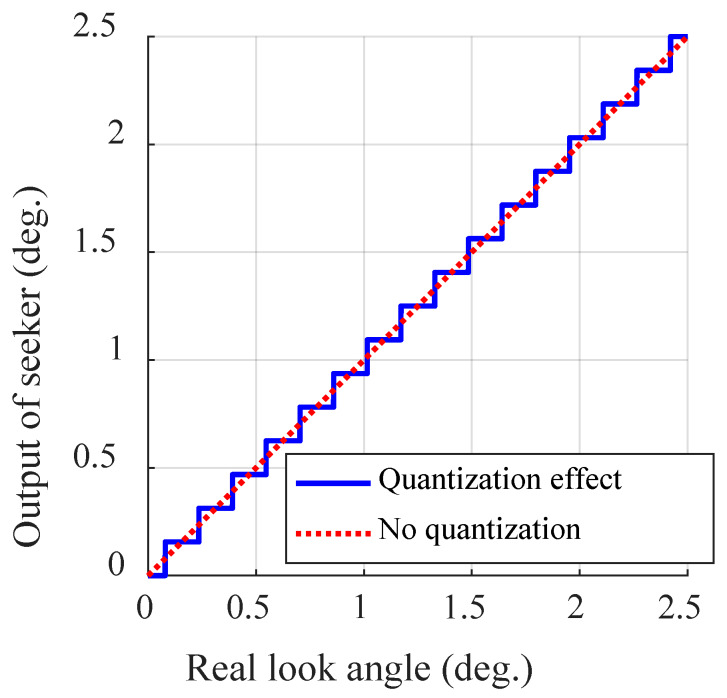
Quantization effect of a seeker.

As an image plane of a strapdown seeker consists of pixels, quantization occurs in a look angle. It is an inherent property as the pixel is a positive integer. This leads to an inevitable loss of target information. The output of the seeker, $\epsilon_{q}$, is determined by its field of view, $S_{fv}$, and resolution, $S_{r}$, as follows:

(12) $$\epsilon_{q}=\left(S_{fv}/S_{r}\right)\mathrm{round}\left(\epsilon /\left(S_{fv}/S_{r}\right)\right),$$

where round() rounds off to the nearest integer. Figure 3 shows $\epsilon_{q}$ according to ϵ, where $S_{fv}$ is $20^{{}^{\circ}}$ and $S_{r}$ is 128.

### 3.3. Target Position Estimator and Flight-Path Angle Construction

The formulated MPC problem requires the flight-path angle of the target for *b*. Usually, it is not easy to measure $\gamma_{t}$, and therefore it is constructed using a look angle obtained by the seeker as a measurement with position estimator. The estimation of the target position is based on discrete-time EKF.

Let us define ${\left[x_{m,k}^{i}\;z_{m,k}^{i}\right]}^{T}$ and ${\left[x_{t,k}^{i}\;z_{t,k}^{i}\right]}^{T}$ as the position vectors of the missile and the target, respectively, in the reference coordinate system, *i*. A discrete-state equation for the estimator can be expressed as follows:

(13) $$\left[\begin{array}{c} x_{t,k+1}^{i}\\ {\dot{x}}_{t,k+1}^{i}\\ z_{t,k+1}^{i}\\ {\dot{z}}_{t,k+1}^{i} \end{array}\right]=\left[\begin{array}{cccc} 1 & \Delta t_{e} & 0 & 0\\ 0 & 1 & 0 & 0\\ 0\; & 0 & 1 & \Delta t_{e}\\ 0 & 0 & 0 & 1 \end{array}\right]\underset{\equiv x_{e,k}}{\underset{︸}{\left[\begin{array}{c} x_{t,k}^{i}\\ {\dot{x}}_{t,k}^{i}\\ z_{t,k}^{i}\\ {\dot{z}}_{t,k}^{i} \end{array}\right]}}+\underset{\equiv w_{e}}{\underset{︸}{\left[\begin{array}{c} w_{x}\\ w_{\dot{x}}\\ w_{z}\\ w_{\dot{z}} \end{array}\right]}},$$

where $\Delta t_{e}$ is a discretization step size of the estimator and $w_{e}∼N\left(O,Q_{e}\right)$ is a process noise. For measurement, the look angle can be modeled utilizing a direction cosine matrix and inverse trigonometric function as follows:

(14) $$y_{e,k}=\tan^{-1}\frac{-\left[\sin\theta_{m}\left(x_{t,k}^{i}-x_{m,k}^{i}\right)+\cos\theta_{m}\left(z_{t,k}^{i}-z_{m,k}^{i}\right)\right]}{\cos\theta_{m}\left(x_{t,k}^{i}-x_{m,k}^{i}\right)-\sin\theta_{m}\left(z_{t,k}^{i}-z_{m,k}^{i}\right)}+v_{e},$$

where quantization of the look angle is treated as a measurement error $v_{e}∼N\left(0,R_{e}\right)$, and $\theta_{m}$ is a pitch angle of the missile.

(15) $$\theta_{m}=\alpha_{m}+\gamma_{m}.$$

Finally, using these state and measurement equations, Equations (13) and (14), the EKF can be designed to estimate the position of the target ${\left[{\hat{x}}_{t,k}^{i}\;{\hat{z}}_{t,k}^{i}\right]}^{T}$. A well-known discrete-time EKF derivation can be found in [38].

Once ${\left[{\hat{x}}_{t,k}^{i}\;{\hat{z}}_{t,k}^{i}\right]}^{T}$ is obtained, the velocity of the target can be computed using the Finite Difference Method (FDM) as follows:

(16) $${\dot{\hat{x}}}_{t,k}^{i}=\frac{{\hat{x}}_{t,k}^{i}-{\hat{x}}_{t,k-1}^{i}}{\Delta t_{e}}\;,\;\;{\dot{\hat{z}}}_{t,k}^{i}=\frac{{\hat{z}}_{t,k}^{i}-{\hat{z}}_{t,k-1}^{i}}{\Delta t_{e}}.$$

Note that ${\left[{\dot{\hat{x}}}_{t,k}^{i}\;{\dot{\hat{z}}}_{t,k}^{i}\right]}^{T}$ from the estimator is not directly used because $y_{e,k}$ in Eqution (14) contains no information on ${\dot{x}}_{t,k}^{i}$ and ${\dot{z}}_{t,k}^{i}$. That is, use of the measurement in state estimate updates poorly affects ${\left[{\dot{\hat{x}}}_{t,k}^{i}\;{\dot{\hat{z}}}_{t,k}^{i}\right]}^{T}$.

Finally, the flight-path angle of the target can be constructed by the following definition:

(17) $${\hat{\gamma }}_{t}=\tan^{-1}\frac{-{\dot{\hat{z}}}_{t,k}^{i}}{{\dot{\hat{x}}}_{t,k}^{i}}.$$

### 3.4. Flight-Path Angle Prediction Using Polynomial Fitting

Since the MPC problem in Equation (10) considers not only the current state but also all the states within the length of the time horizon, the flight-path angle of the target for each node should be allocated. Polynomial curve fitting is applied in a least-squares sense regarding this matter [36].

As shown in Figure 1, the relationship between the flight-path angle and the acceleration of the target can be described as follows:

(18) $${\dot{\gamma }}_{t}=\frac{a_{t}}{v_{t}}.$$

Since a pull-up maneuver is considered, the target acceleration varies linearly. With this consideration, the flight-path angle follows a polynomial function of degree 2 with respect to time.

(19) $$p\left(t\right)=p_{1}t^{2}+p_{2}t+p_{3}.$$

If the target maneuvers with nonzero constant acceleration, the polynomial fitting algorithm will return $p_{1}≃0$. Although constant acceleration may be witnessed during the pull-up maneuver, the case of linear acceleration also exists. To include both cases, a polynomial of degree 2 is appropriate to be considered in this paper for the flight-path angle prediction.

For least-squares fitting, $\hat{\gamma_{t}}$ from the estimator should be continuously collected from the beginning of terminal homing: $\left(t^{1},{\hat{\gamma }}_{t,1}\right)$, $\left(t^{2},{\hat{\gamma }}_{t,2}\right)$, ⋯, $\left(t^{L},{\hat{\gamma }}_{t,L}\right)$. Then, the following problem is solved for polynomial coefficients $p_{1}$, $p_{2}$, and $p_{3}$ at every iteration of MPC.

(20) $$\begin{array}{cccc} \; & \underset{p_{1},p_{2},p_{3}}{\mathrm{minimize}} & \; & \sum_{\ell =1}^{L}{\left({\hat{\gamma }}_{t,\ell }-p\left(t^{\ell }\right)\right)}^{2}\;, \end{array}$$

where $p(t^{\ell })$ is expressed in Equation (19).

To apply flight-path angle prediction, let us assume that the current time is $t^{\ell }$ and the MPC process is about to begin. Compared to the previous MPC problem in Equation (10), *b* is replaced by $b_{k}$, which is defined in a similar way as follows:

(21) $$b_{k}=\left(\int_{O}^{\Delta t_{c}}e^{\tau \bar{A}}\right){\bar{b}}_{k},$$

(22) $${\bar{b}}_{k}={\left[\begin{array}{ccccc} 0 & 0 & 0 & 0 & v_{t}\sin{\hat{\gamma }}_{t}\left(k\right) \end{array}\right]}^{T},$$

where ${\hat{\gamma }}_{t}\left(k\right)$ can be obtained by the following equation.

(23) $${\hat{\gamma }}_{t}\left(k\right)=p\left(t^{\ell }+\left(k-1\right)\Delta t_{c}\right).$$

## 4. Numerical Simulation

### 4.1. Single-Run Simulation

Numerical simulation is performed to demonstrate the performance of the proposed integrated guidance and control method. The total engagement time is 3.0 s, and $\lambda_{0}=0^{{}^{\circ}}$. The parameters of the missile [17,32] and its MPC are listed in Table 1.

In Table 1, *g* is a standard gravity (≡ 9.8067 m/s${}^{2}$) and diag is a square diagonal matrix with the elements inside the square brackets on the main diagonal. The constant target speed, $v_{t}$, is set to 380 m/s. The target acceleration, $a_{t}$, and the initial value of $\gamma_{t}$ are chosen as −3.5 g and $175^{{}^{\circ}}$, respectively, which are unknown variables. The initial-state variable of the missile is set to [0${}^{{}^{\circ}}$ 0${}^{{}^{\circ}}$ 0${}^{{}^{\circ}}$/s 5${}^{{}^{\circ}}$ 50 m]${}^{T}$, where the order of the state is shown in Equation (5).

For the MPC problem in Equation (10), a nonzero number is assigned to the fifth diagonal element in $Q_{k}$ so that a small $z_{m}$ is to be achieved for intercepting the target, as shown in Table 1. The other diagonal elements in $Q_{k}$ are left at zero because they are not required to be regulated. The optimization problem is solved by primal–dual interior-point method [36] in this study.

The performance using flight-path angle prediction is compared with the case of no prediction. In the no prediction case, MPC is computed with the current estimated flight-path angle, instead of Equation (23), as follows:

(24) $${\hat{\gamma }}_{t}\left(k\right)={\hat{\gamma }}_{t,\ell }.$$

**Figure 4:**
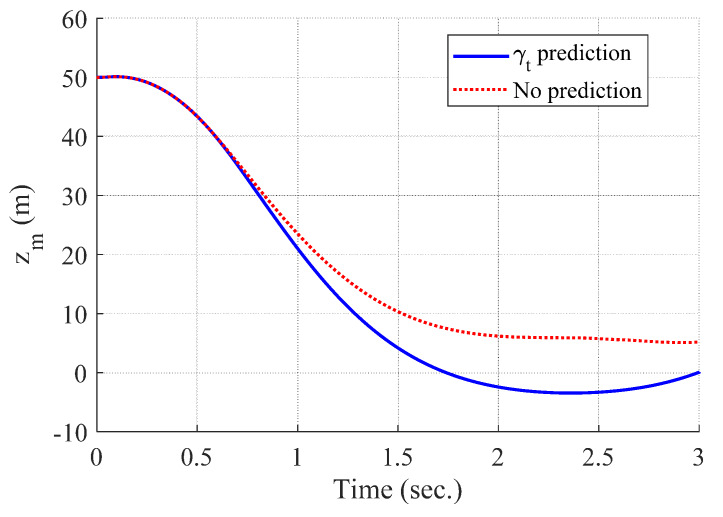
Relative displacement between the target and the missile (single-run simulation).

**Figure 5:**
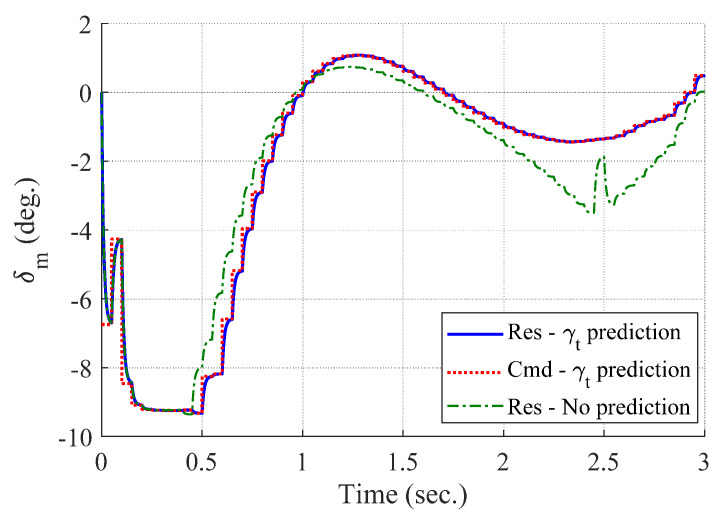
Control input of the missile (single-run simulation).

**Figure 6:**
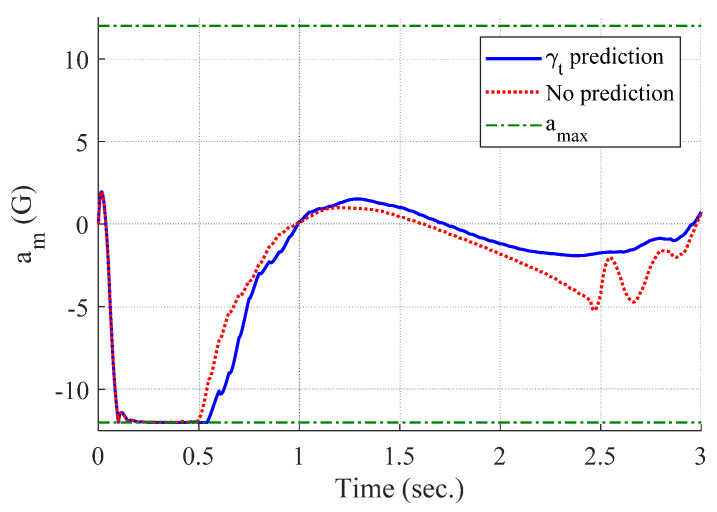
Acceleration of the missile (single-run simulation).

**Figure 7:**
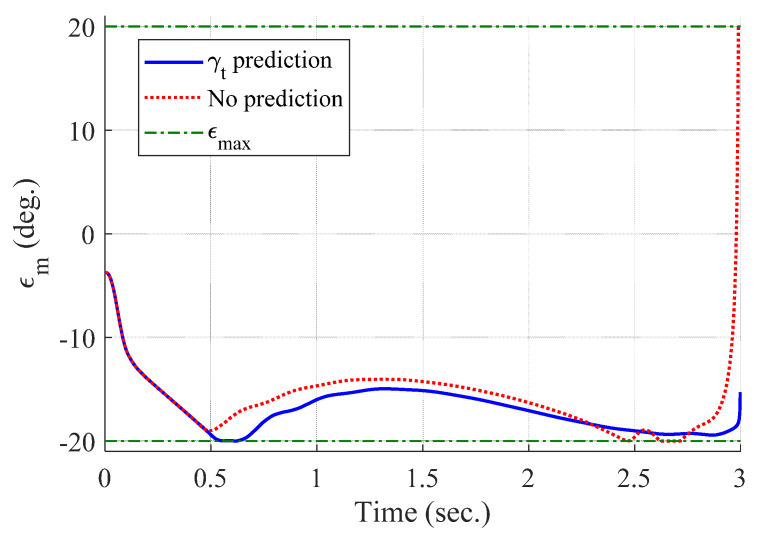
Look angle of the missile (single-run simulation).

Figure 4 and Figure 5 show the time responses of the relative displacement and the control input, respectively. The final miss distance of the proposed algorithm is 0.0668 m, whereas that of MPC without prediction is 5.1782 m. Command $\delta_{c}$ obtained from MPC (dotted line in Figure 5) is produced at every $\Delta t_{c}$ and stays the same for the next $\Delta t_{c}$, as shown in Figure 2. Figure 6 and Figure 7 show the time responses of the acceleration and the look angle of the missile, respectively. Each variable stays within the predefined limit during the entire engagement, which means that the formulated MPC problem gives the feasible solution satisfying the inequality constraints.

**Figure 8:**
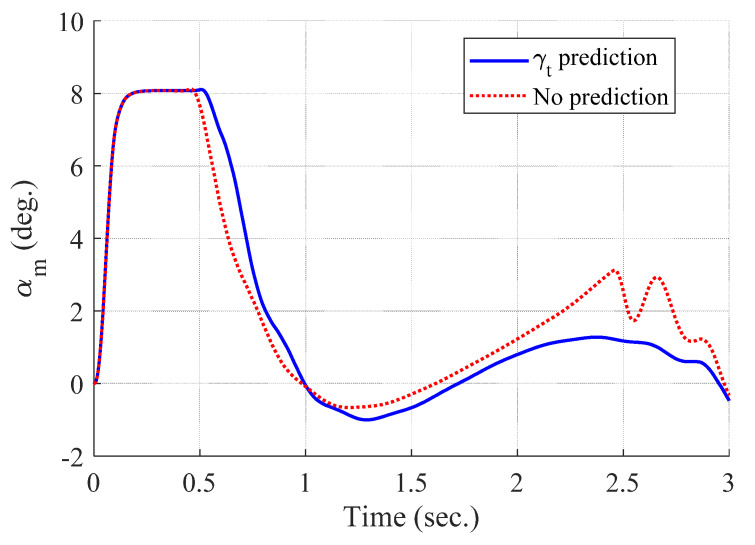
Angle of attack of the missile (single-run simulation).

**Figure 9:**
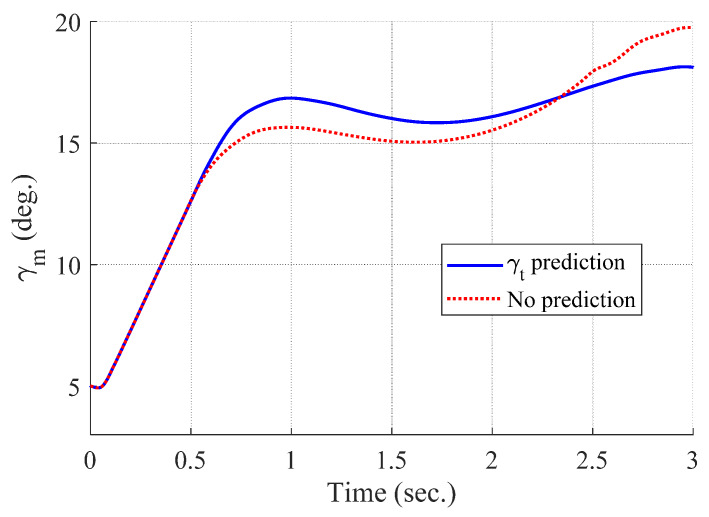
Flight-path angle of the missile (single run-simulation).

**Figure 10:**
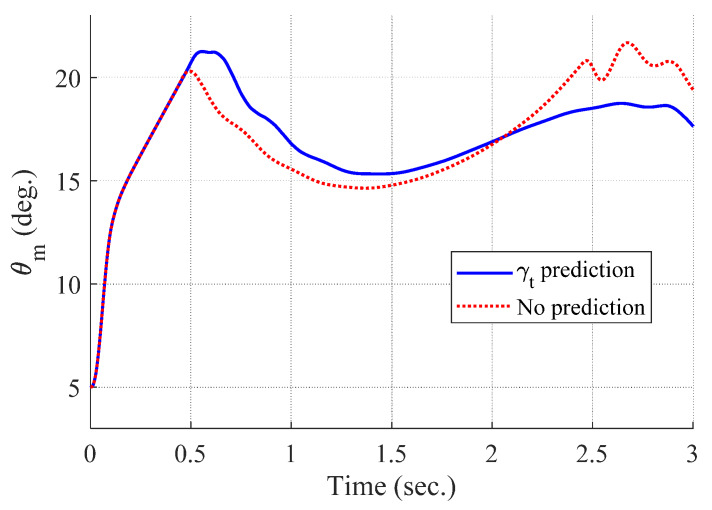
Pitch angle of the missile (single-run simulation).

**Figure 11:**
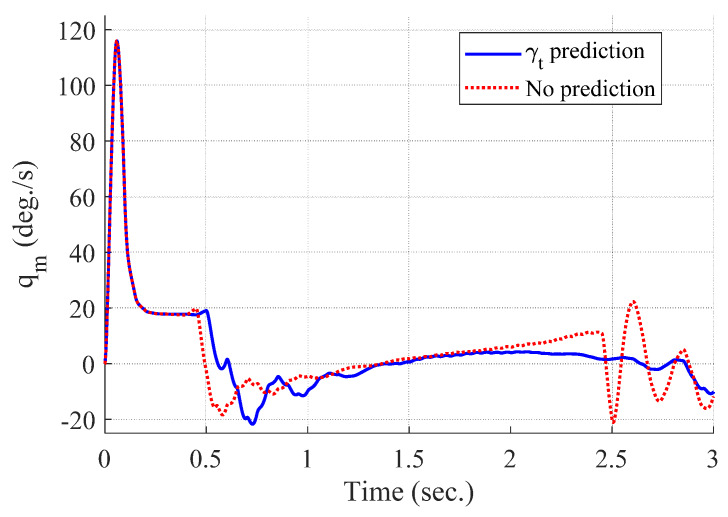
Pitch rate of the missile (single-run simulation).

Figure 8, Figure 9, Figure 10 and Figure 11 show the time responses of the angle of attack, the flight-path angle, the pitch angle, and the pitch rate of the missile, respectively. Without flight-path angle prediction, the missile cannot handle the target’s pull-up maneuver as time goes on, and MPC gives the solution that oscillates at the end of terminal homing, as shown in Figure 5. This results in a poor miss distance as well as unnecessary acceleration effort as shown in Figure 4 and Figure 6. The oscillatory phenomenon can also be found in Figure 8, Figure 9, Figure 10 and Figure 11. Furthermore, divergence of the look angle at the end of terminal homing can be found with the no prediction case, as shown in Figure 7.

The computation time of different sampling intervals and time horizon lengths is listed in Table 2. The proposed algorithm is conducted using Matlab R2019b software with Intel Core i9-9900K CPU running at 3.60 GHz and 32.0 GB RAM in a 64-bit operating system. Table 2 shows the average time for solving a single MPC problem for 10 runs for each case. It is shown that the computation time depends on *N* ($=t_{c}/\Delta t_{c}$), which is the number of samples. Note that computational complexity for the proposed algorithm mainly comes from solving the convex optimization problem in Equation (10), of which the problem size grows linearly with *N*. Also, note that Equation (10) constitutes a sparse quadratic programming problem, and the computational complexity for solving sparse quadratic programming via the interior point methods with sparse matrix factorization is proportional to the number of nonzero elements in the associated KKT matrix, which is proportional to the problem size [36,37]. This matches very well with the results observed in Table 2.

### 4.2. Monte Carlo Simulation

A Monte Carlo simulation is carried out to investigate the robustness of the proposed algorithm. A total of 300 different cases are generated where target missile properties and interceptor’s initial states are chosen at random using a normal distribution. The range of initial $\gamma_{t}$ is set to $\left[150^{{}^{\circ}},\;210^{{}^{\circ}}\right]$, and the acceleration of the target varies with respect to time as follows:

(25) $$a_{t}=a_{t1}t+a_{t2},$$

where the ranges of $a_{t1}$ and $a_{t2}$ are set to [−1 g/s, 1 g/s] and [−4 g, 4 g], respectively. Also, the ranges of initial $\delta_{m}$, $\alpha_{m}$, $q_{m}$, $\gamma_{m}$, and $z_{m}$ are set to $\left[-2^{{}^{\circ}},\;2^{{}^{\circ}}\right]$, $\left[-5^{{}^{\circ}},\;5^{{}^{\circ}}\right]$, $\left[-10^{{}^{\circ}}/s,\;10^{{}^{\circ}}/s\right]$, $\left[-10^{{}^{\circ}},\;10^{{}^{\circ}}\right]$, and [−100 m, 100 m], respectively. Note that the above eight random variables are fixed for each scenario. For the other parameters, the same values from the single-run simulation are applied. Furthermore, infeasible scenarios due to the random initial condition are excluded from the result.

**Figure 12:**
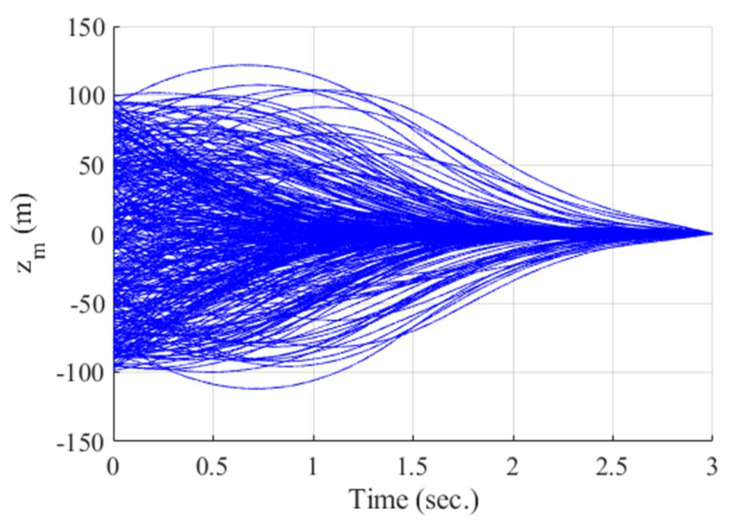
Relative displacement between the target and the missile (Monte Carlo simulation).

**Figure 13:**
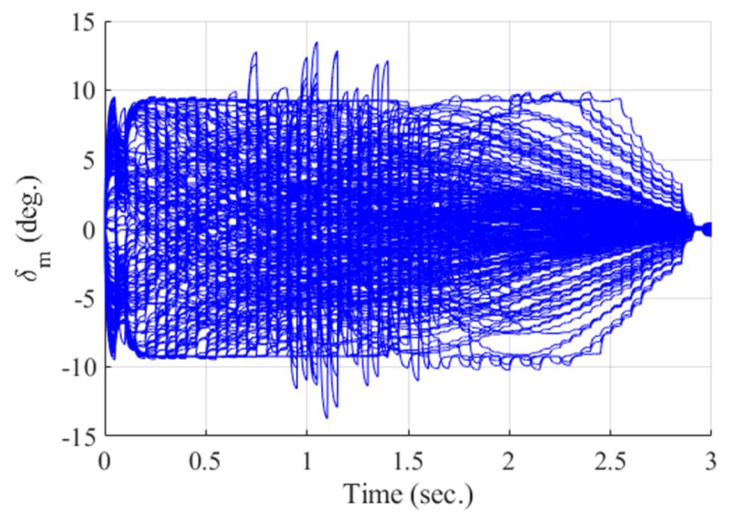
Control input of the missile (Monte Carlo simulation).

**Figure 14:**
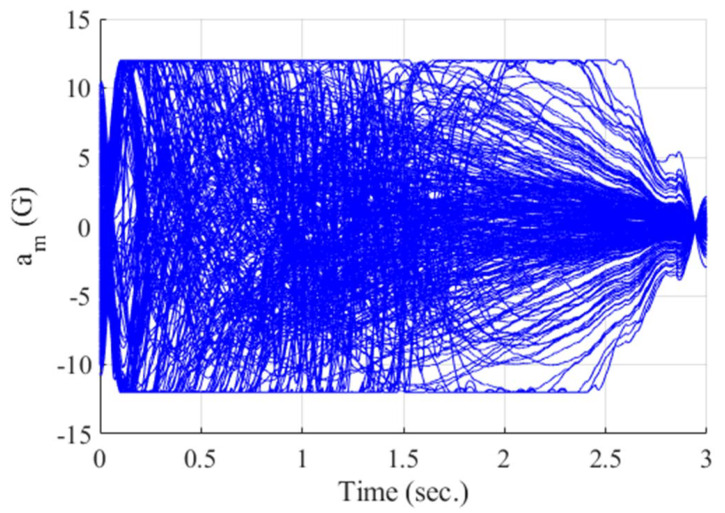
Acceleration of the missile (Monte Carlo simulation).

**Figure 15:**
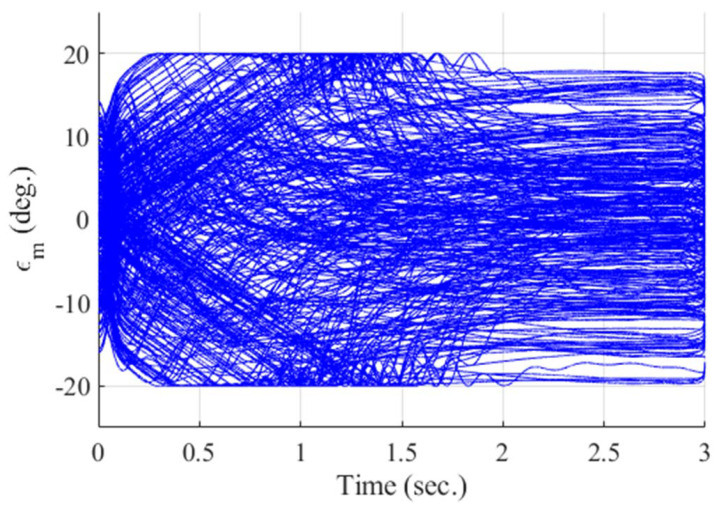
Look angle of the missile (Monte Carlo simulation).

**Figure 16:**
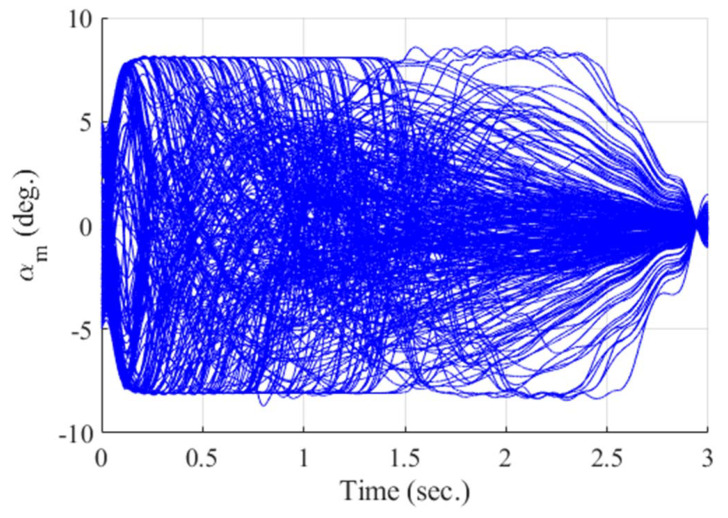
Angle of attack of the missile (Monte Carlo simulation).

**Figure 17:**
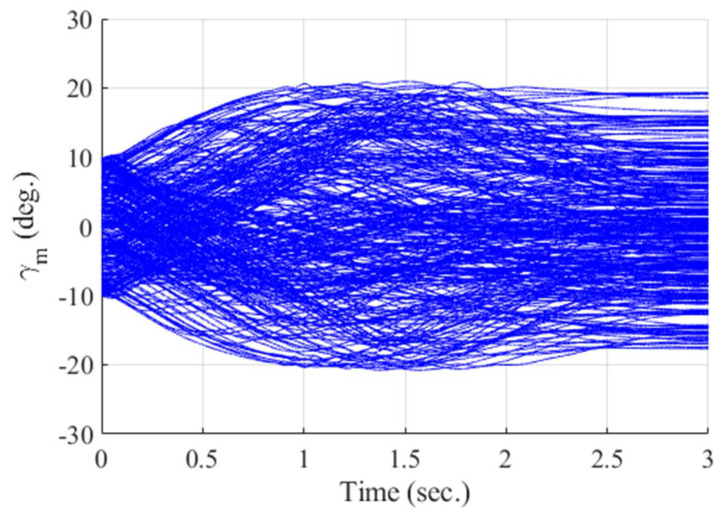
Flight-path angle of the missile (Monte Carlo simulation).

**Figure 18:**
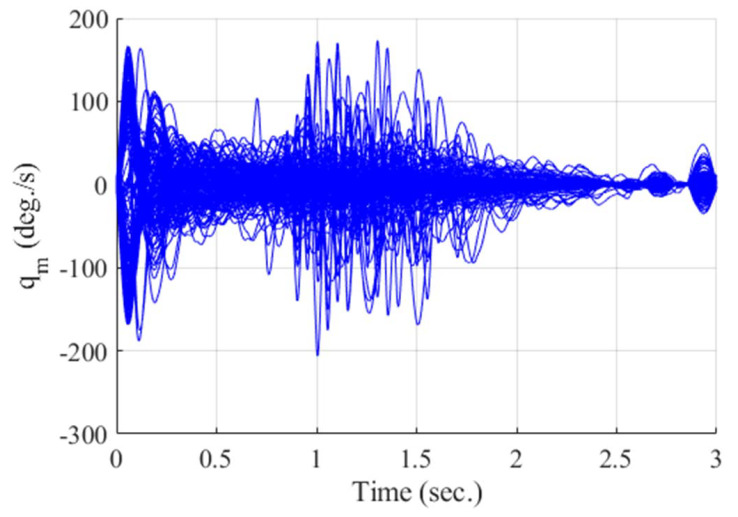
Pitch rate of the missile (Monte Carlo simulation).

Figure 12, Figure 13, Figure 14, Figure 15, Figure 16, Figure 17 and Figure 18 show the time responses of the relative displacement, the control input, the acceleration, the look angle, the angle of attack, the flight-path angle, and the pitch rate of the missile, respectively. As shown in Figure 12, the final miss distances are all within 0.2 m, which can be interpreted as a direct hit. In most cases, the actuator of the missile operates in the range of $\left[-10^{{}^{\circ}},\;10^{{}^{\circ}}\right]$, as demonstrated in Figure 13. However, the missile uses its actuator up to $\left[-14^{{}^{\circ}},\;14^{{}^{\circ}}\right]$ in some scenarios with a cold initial condition. Furthermore, a small $\delta_{m}$ can be witnessed at the end of terminal homing. Since $\delta_{m}$ is produced with the consideration of $\gamma_{t}$ prediction, the future maneuver of the target is resolved in $u_{0}^{*}$. This results in no overload on the actuator at the end of terminal homing. On the other hand, the acceleration and the look angle of the missile are within the predefined limit, 12 g and $20^{{}^{\circ}}$, for all scenarios, as shown in Figure 14 and Figure 15. Since the angle of attack, the flight-path angle, and the pitch rate of the missile are not regulated by $Q_{k}$ in the formulated MPC problem, their final values are different in each scenario in Figure 16, Figure 17 and Figure 18.

## 5. Conclusions

In this study, integrated guidance and control based on MPC for terminal homing in two-dimensional space is proposed. The short-period dynamics as well as actuator dynamics is considered in equations of motion. The optimization problem in MPC is solved by the primal–dual interior-point method, and the first element of the achieved control input is applied to the missile system. This is an iterative process until interception. On the other hand, the look angle from the strapdown seeker with an inherent quantization effect is used to build the EKF. With the estimated target position, the flight-path angle is constructed using FDM. Then, a polynomial algorithm is performed with collected flight-path angle information to generate polynomial coefficients. A polynomial of degree 2 is adopted as a pull-up maneuvering target is considered in this study. These coefficients are used in MPC for future nodes within the time horizon. The numerical simulation demonstrates that flight-path angle prediction plays an important role at the end of terminal homing. Also, a Monte Carlo simulation result verifies the robustness of the proposed algorithm in terms of the target’s maneuver and the interceptor’s initial state.

For future research, the proposed integrated guidance and control algorithm will be extended to deal with a weaving maneuver. Also, MPC is generally known for its high computational cost. Therefore, an effective design approach will be investigated for the real-time implementation of the proposed MPC.

## Figures and Tables

**Table 1:** Parameters of the missile and its MPC.

Missile			
$Z_{\alpha }$	−2.9399 s${}^{-1}$	$v_{m}$	380 m/s
$Z_{\delta }$	−0.6497 s${}^{-1}$	$a_{max}$	12 g
$M_{\alpha }$	−623.6149 s${}^{-2}$	$\epsilon_{max}$	$20^{{}^{\circ}}$
$M_{q}$	−5 s${}^{-1}$	$S_{fv}$	$20^{{}^{\circ}}$
$M_{\delta }$	−554.4808 s${}^{-2}$	$S_{r}$	256
$\omega_{a}$	100 s${}^{-1}$		
MPC			
$t_{c}$	3 s		
$\Delta t_{c}$	0.05 s		
$Q_{k}$	$diag[0\;0\;0\;0\;7e^{-3}]$	for k∈{0,⋯,N}	
$R_{k}$	1	for k∈{0,⋯,N−1}	

**Table 2:** Computation time of the proposed MPC problem.

	$t_{c}$	1.00	1.50	2.00	2.50	3.00
$\Delta t_{c}$						
0.01		0.0314	0.0517	0.0713	0.0946	0.1211
0.05		0.0067	0.0101	0.0134	0.0168	0.0206
0.10		0.0038	0.0055	0.0070	0.0086	0.0106
